# A Case of Malposition of the Fœtus Sixty Hours in Labor

**Published:** 1881-10-20

**Authors:** Wm. T. Beall

**Affiliations:** Mississippi


### A CASE OF MALPOSITION OF THE FŒTUS SIXTY HOURS IN LABOR.

BY WM. T. BEALL, M. D., OF MISSISSIPPI.

If you find the following worth publication, please give it a place in your Journal:

On the morning of the 11th of March I was called up at 5 o'clock to see a woman in labor. Found a bright mulatto aged about 45 or 46, the mother of 20 children, in great pain and nearly exhausted, having been taken with labor pains 60 hours before, and two old granny women sitting by her expecting to receive the child with each pain. Upon inquiry I found the waters had escaped some 24 hours previous to my arrival. I asked the attendants if they had made an examination to see what was the difficulty? They said they had, and everything was right.

Upon examination, I found the left arm folded upon the shoulder, and the child laying across the brim of the pelvis with the head in the right side. Introducing my right hand into the womb and finding it resistless, having nearly exhausted itself, I straightened the arm, laying it along side the body, took the head in the palm of my hand, raised the fœtus up and brought the head in position, withdrew my hand, gave a large dose of extract of ergot, and upon the appearance of pain gave chloroform. In two hours after my arrival she was delivered of a male child weighing ten pounds, but still-born, requiring half hour's work to bring it to active life.

Here is a case, Mr. Editor, of a woman having given birth to 20 children, several times having twins, and never having need for a physician (for this is the first time she ever called in a doctor to attend her in labor), and in giving birth to her 21st would have lost her life without timely aid. As it was, she very narrowly escaped, for she could not have survived much longer had not something been done to correct the mal-presentation.

